# Cerebral Congestion

**Published:** 1888-03

**Authors:** William A. Hawley

**Affiliations:** Syracuse, N. Y.


### CEREBRAL CONGESTION.

William A. Hawley, M. D., Syracuse, N. Y.

The case was one of cerebral congestion, resulting from sunstroke and intense overwork, in a stout, plethoric man of about fifty. It had yielded to Bry., so that I thought he would be out in a day or two, but company and some business fatigued him so that he was very restless over night, and got uncovered, taking cold. The next morning he did not seem so well as the day before, but I gave him only S. L. The second evening, as I went in, he said, "O Doctor! I have been in hell all night, and have not slept a wink." He had a facial neuralgia, left side, so severe he could not keep still, darting, shooting, flashing pains, that come and go like lightning. I dissolved a little Spong.$^{cc}$, and directed a teaspoonful every half hour till relieved, and then stop, promising to be in again after dinner. I was there at two o'clock, when he said, "Doctor, I took that medicine exactly according to directions till twelve, but every dose aggravated those pains till I could not stand it, and I have taken none since." I gave S. L. in water every half hour, and called again about five. Found him quiet and free from pain, but afraid to move lest it come on again. He informed me that he *must* have something to make him quiet and make him sleep that night. Continued the same. At nine o'clock found he had slept an hour and was free from pain, but he said, "Doctor, I have got to have an anæsthetic to-night; I cannot go through another night so." I said, "I suppose you do not intend to dictate to the doctor?" "Oh! no." "Well, I will fix that," and then I called for another glass of water, and put a powder of S. L. in it and directed that he take two spoonfuls once an hour till he slept. It ended the case, and he has returned to his home in New York. He is a client of T. F. Allen's. He and his wife were greatly pleased with the result.

